# The Efficacy of HGF/VEGF Gene Therapy for Limb Ischemia in Mice with Impaired Glucose Tolerance: Shift from Angiogenesis to Axonal Growth and Oxidative Potential in Skeletal Muscle

**DOI:** 10.3390/cells11233824

**Published:** 2022-11-29

**Authors:** Iurii S. Stafeev I, Maria A. Boldyreva, Svetlana S. Michurina, Margarita Yu. Agareva, Arina V. Radnaeva, Mikhail Yu. Menshikov, Yu-Chen Hu, Pavel I. Makarevich, Yelena V. Parfyonova

**Affiliations:** 1 National Medical Research Centre for Cardiology Named after Academician E.I.Chazov, 121552 Moscow, Russia; 2 Cell and Molecular Biotechnology Unit, Faculty of Biology and Biotechnology, National Research University Higher School of Economics, 101000 Moscow, Russia; 3 Faculty of Biology, Lomonosov Moscow State University, 117192 Moscow, Russia; 4 Institute of Fine Chemical Technologies Named after M.V. Lomonosov, 119571 Moscow, Russia; 5 Faculty of Medicine, Lomonosov Moscow State University, 119192 Moscow, Russia; 6 Institute for Regenerative Medicine, Medical Research and Education Centre, Lomonosov Moscow State University, 119192 Moscow, Russia; 7 Department of Chemical Engineering, National Tsing Hua University, Hsinchu 300044, Taiwan; 8 Frontier Research Center on Fundamental and Applied Sciences of Matters, National Tsing Hua University, Hsinchu 300044, Taiwan

**Keywords:** gene therapy, plasmid, limb ischemia, high-fat diet, obesity, VEGF, HGF

## Abstract

Background: Combined non-viral gene therapy (GT) of ischemia and cardiovascular disease is a promising tool for potential clinical translation. In previous studies our group has developed combined gene therapy by vascular endothelial growth factor 165 (*VEGF165*) + hepatocyte growth factor (*HGF*). Our recent works have demonstrated that a bicistronic pDNA that carries both human *HGF* and *VEGF165* coding sequences has a potential for clinical application in peripheral artery disease (PAD). The present study aimed to test *HGF/VEGF* combined plasmid efficacy in ischemic skeletal muscle comorbid with predominant complications of PAD-impaired glucose tolerance and type 2 diabetes mellitus (T2DM). Methods: Male C57BL mice were housed on low-fat (LFD) or high-fat diet (HFD) for 10 weeks and metabolic parameters including FBG level, ITT, and GTT were evaluated. Hindlimb ischemia induction and plasmid administration were performed at 10 weeks with 3 weeks for post-surgical follow-up. Limb blood flow was assessed by laser Doppler scanning at 7, 14, and 21 days after ischemia induction. The necrotic area of *m.tibialis anterior*, macrophage infiltration, angio- and neuritogenesis were evaluated in tissue sections. The mitochondrial status of skeletal muscle (total mitochondria content, ETC proteins content) was assessed by Western blotting of muscle lysates. Results: At 10 weeks, the HFD group demonstrated impaired glucose tolerance in comparison with the LFD group. *HGF/VEGF* plasmid injection aggravated glucose intolerance in HFD conditions. Blood flow recovery was not changed by *HGF/VEGF* plasmid injection either in LFD or HFD conditions. GT in LFD, but not in HFD conditions, enlarged the necrotic area and CD68+ cells infiltration. However, *HGF/VEGF* plasmid enhanced neuritogenesis and enlarged NF200+ area on muscle sections. In HFD conditions, *HGF/VEGF* plasmid injection significantly increased mitochondria content and ETC proteins content. Conclusions: The current study demonstrated a significant role of dietary conditions in pre-clinical testing of non-viral GT drugs. *HGF/VEGF* combined plasmid demonstrated a novel aspect of potential participation in ischemic skeletal muscle regeneration, through regulation of innervation and bioenergetics of muscle. The obtained results made *HGF/VEGF* combined plasmid a very promising tool for PAD therapy in impaired glucose tolerance conditions.

## 1. Introduction

Combined non-viral gene therapy (GT) for limb ischemia and other cardiovascular disorders has emerged due to numerous unsuccessful clinical trials of GT drugs that use only one gene [1]. In most cases, potent angiogenic growth factor or cytokine has been delivered to human tissues via GT using naked plasmid DNA (pDNA) injected to induce transient local protein production in ischemic tissue [2,3]. However, phase II-III clinical trials in patients with peripheral artery disease (PAD), myocardial infarction or ischemic heart failure have demonstrated high tolerability of pDNA GT drugs along with modest or no efficacy signs [4,5,6].

In an attempt to overcome this obstacle, our group has developed and investigated combined gene therapy by vascular endothelial growth factor 165 (VEGF165) + hepatocyte growth factor (HGF) delivered as a mixture of pDNAs [7] or in a single bicistronic vector [8]. During the last decade we have demonstrated superiority of this approach over sole gene delivery of either *VEGF165* or *HGF*. We also elaborated on pleiotropic effects of *VEGF+HGF* including regulation of inflammation (in particular, taxis of monocytes) and stabilization of hypoxia-inducible factors (HIFs) that play a pivotal role in the regulation of sprouting and blood vessel maturation [7].

Our recent works have demonstrated that a bicistronic pDNA that carries both human *HGF* and *VEGF165* coding sequences has a potential for clinical translation for treatment of PAD in humans [8]. We also focused on p*HGF/VEGF* due to its higher druggability and ease of pharmacological development using a single pDNA rather than a mixture of several vectors with barely predictable kinetics [7,9].

Prior to further advance, we tried to understand putative caveats that potentially hindered the above mentioned clinical trials and focused on the relevance of pre-clinical models used to assess pDNA efficacy in PAD. We have decided to take into account the most frequent metabolic comorbidities of PAD. They may include, yet are not limited to, obesity, metabolic syndrome, glucose tolerance disorders and type 2 diabetes mellitus (T2DM), widespread in patients suffering from limb ischemia. Obesity significantly increases the risk of PAD development and comorbidity of T2DM accounts for up to 30–40% of patients primarily diagnosed with PAD known to be associated with worsened prognosis and higher amputation risks [10,11,12].

Thus, evaluation of gene therapy preclinical efficacy requires an animal model that would provide a pathophysiological setting that would reproduce factors and conditions influencing potential benefits in humans. Induction of glucose tolerance impairment and/or T2DM in rodent and non-rodent species can be achieved using genetic-based strains [13,14], specific surgery [13,15] or by alimentary interventions using high-fat chows [13,16]. The latter is a more clinically relevant model although it may take substantial time (8 to 36 weeks) to significantly alter body weight, insulin sensitivity and fasting blood glucose (FBG) level to mimic the corresponding disorder [17,18,19]. We used a diet-based mouse model of glucose tolerance impairment described and adopted in published studies [20,21,22] and investigated its impact on *HGF/VEGF* efficacy in mice with hindlimb ischemia to gain insight into systemic metabolic changes and skeletal muscle bioenergetics. Previously our group has demonstrated that *HGF/VEGF* induced angiogenesis in non-diabetic mice consuming standard grain-based chow [8]. The current work is a contribution to the field that would help in development and preclinical assessment of the pharmacological efficacy of non-viral (mainly, pDNA-based) GT drugs to treat PAD.

## 2. Methods and Materials

### 2.1. Animal Strain, Housing, Diets and Ethical Approval

Five-week old male mice (C57/B6 strain) were purchased from “Andreevka” animal husbandry facility (Moscow region, Russia). After 5 days of acclimation, mice were matched by body weight (17–23 g), fasting blood glucose (FBG) level (3.9–6.9 mM) and randomized to either low fat diet (LFD) or high fat diet (HFD) group (n = 14–16 per group). During the next 10 weeks animals were kept under specific pathogen-free conditions with chow and water ad libitum and regular 12:12 light–dark cycle. Both low-fat diet (LFD) (#D12450H) and high-fat diet (HFD) (#D12451) chows were purchased from “Research Diets”, USA. During the experiment, the body weight, FBG and food consumption were measured every 2 weeks. For the FBG measurements, we used a Contour Plus One glucometer (Ascensia Diabetes Care, Basel, Switzerland). At 10 weeks, all mice underwent surgical modeling of hindlimb ischemia. Experimental protocols including surgical manipulations and euthanasia were designed in accordance to Institute and National regulations. All animal experiments were carried out in compliance with internal “Rules for conducting work using experimental animals” and were approved by the Ethical Board of the National Medical Research Center of Cardiology (permit # 16-10-00).

### 2.2. Glucose and Insulin Tolerance Tests

Glucose tolerance test (GTT) and insulin tolerance test (ITT) were performed at 10 weeks of dietary intervention before surgery and at 13 weeks (10 weeks of diets + 3 weeks after surgery and plasmid injection). Before both tests, mice underwent overnight fasting and body weight measurement. Peripheral blood samples were collected from the tail vein after dissection by a sterile scalpel; glucose level was assayed by a glucometer. For the GTT, 2 g/kg of glucose (10% D-glucose solution, Sigma-Aldrich, Burlington, MA, USA) was injected intraperitoneally. Glucose levels were measured before injection and at 15, 30, 45, 60, 90 and 120 min after injection. For ITT, 0.5 IU/kg of human insulin (Humulin, Lilly France GmbH, Paris, France) was injected intraperitoneally. Glucose levels were measured before injection and at 15, 30, 45, 60, 90, 120, 150 and 180 min after injection.

### 2.3. Plasmid Vector Design and Purification

Design of bicistronic pDNA carrying coding sequences of human *HGF* and *VEGF165* is described in detail in our previous work [7]. *HGF/VEGF* pDNA was amplified in *Escherichia coli* (DH-5α strain) and purified to endotoxin-free grade using the EndoFree Plasmid Giga Kit (Qiagen, Germantown, MD, USA) as recommended by the manufacturer, followed by reconstitution in sterile saline (0.9% NaCl) for animal administration.

### 2.4. Hindlimb Ischemia Modeling and Postsurgical Care

At 10 weeks of dietary intervention, mice were narcotized by intraperitoneal injection of 2.5% avertin solution. All surgical manipulations were carried out in aseptic conditions under a binocular microscope Leica M620 TTS (Leica Microsystems, Wetzlar, Germany). Unilateral induction of hindlimb ischemia was performed as previously described [8]. Briefly, skin was dissected by the midline of the left hindlimb and the femoral artery with its branches was isolated and ligated between its proximal part and popliteal bifurcation; the sciatic nerve remained intact during these manipulations. At this step LFD and HFD groups were randomized into subgroups saline (three equal PBS injections, 50 μL per injections; intramuscular injections were performed during hind limb ischemia modeling in the skeletal muscle of thigh middle third) and *HGF/VEGF* (three equal *HGF/VEGF* plasmid solution injections, 50 μL per injections, solution concentration 1mg/mL; intramuscular injections were performed during hind limb ischemia modeling in the skeletal muscle of thigh middle third), n = 7–8 animals per subgroup. After the skin was closed by 5-0 silk sutures, the animals were acclimated in a heated room until full recovery. All animals received 1.5 mL PBS subcutaneous bolus injection for the recovery of water-salt balance and compensation of blood loss. In the post-surgical period, dietary interventions and housing conditions remained unchanged. Laser Doppler perfusion measurements of hindlimb blood flow were performed immediately after surgery to study ischemia onset (0 days) and at days 7, 14 and 21. At week 13, we carried out postsurgical GTT and ITT as described above and mice were sacrificed by lethal isoflurane inhalation; skin was dissected and samples of *m.tibialis anterior* were collected and frozen in TissueTek medium (Sakura Finetek, Torrance, CA, USA) in liquid nitrogen.

### 2.5. Laser Doppler Perfusion Measurement

Laser Doppler scanner MOOR LDI2 (Moor Instruments Ltd., Axminster, UK) was used for subcutaneous blood flow recovery assessment. Animals were narcotized by avertin intraperitoneal injection as described above and perfusion measurements were performed (n = 3–4) on the plantar surfaces of the animal’s feet; variability of the data was analyzed by “Moor Image Review” software. The obtained results were normalized on blood flow in the intact hind limb and are presented as relative perfusion (%). Sequential scans were performed until three consequent measurements with minimal (<10%) deviation.

### 2.6. Hematoxylin/Eosin Staining

Tissue sections were heated to room temperature and fixed in 4% formaldehyde solution with consequent 3 times washing by PBS and rinsed by distilled water. Staining by Mayer’s hematoxylin was performed (5 min) and the dye was differentiated in tap water (1 min). Slides were washed by distilled water and stained by eosin B solution (5 min). After that, slides were washed by 70% ethanol and incubated subsequently in 96% ethanol (2 × 5 min), 100% ethanol (2 × 10 min), xylene (2 × 10 min). Stained sections were mounted in Cytoseal-60 medium (Richard-Allen Scientific, Kalamazoo, MI, USA) under coverslips. Microphotographs were obtained on Leica ScanScope CS (Leica Microsystems, Wetzlar, Germany) scanning microscope and analyzed in Aperio ImageScope (Leica Microsystems) and ImageJ (version 1.53c, Wayne Rasband, NIH, Cambridge, MA, USA) software.

### 2.7. Immunofluorescent Staining

Tissue sections were heated to room temperature and fixed in 4% formaldehyde solution with consequent 3 times washing by PBS. In the next step, the slides were blocked by 10% secondary antibody’s donor serum in PBS supplemented by 2% bovine serum albumin (BSA). Stainings by primary antibodies were performed at 4 °C overnight, using anti-CD68 (#137001, BioLegend, San Diego, CA, USA), anti-CD31 (#553370, BD Biosciences Pharmingen, Franklin Lakes, NJ, USA) or anti-NF200 (#4142, Sigma-Aldrich) anti-mouse antibodies at dilutions specified by the manufacturers. After incubation with primary antibodies and PBS washing, slides were stained by the respective secondary antibodies conjugated with AlexaFluor488 (#A21206, Thermo Scientific, Waltham, MA, USA) and AlexaFluor594 (#A21206, Thermo Scientific). Nuclei were counterstained by DAPI (Sigma-Aldrich) for 5 min and slides were washed by PBS and mounted in medium Aqua Polymount (Polysciences Inc., Warrington, PA, USA) under coverslips. Staining was visualized by fluorescent microscope Zeiss Axiovert A1 (Zeiss, Oberkochen, Germany) and images were analyzed in Axiovision 3.1 (Zeiss) and ImageJ (NIH, USA) software.

### 2.8. Western Blotting

Skeletal muscle samples from *m. quadriceps femoris* were homogenized by a household drill homogenizer (Metabo, Nurtingen, Germany) equipped with micropestles (Eppendorf, Hamburg, Germany) in 1 μL of RIPA buffer per 1 mg of tissue, supplemented with inhibitors of proteases (“cOmplete Tablets”, Roche, Basel, Switzerland) and phosphatases (10 mM sodium glycerophosphate, 20 mM sodium pyrophosphate, 10 mM sodium fluoride, 1 mM sodium orthovanadate). Extracts were centrifuged and supernatants were obtained and mixed with corresponding volumes of 4× sample buffer. After that, extracts were heated to 56 °C (20 min) and separated by Laemmli SDS-PAGE. Proteins were transferred from gel to PVDF membrane; they were blocked by 5% of fat-free milk solution on TBST and incubated with primary and secondary antibodies according to manufacturer’s instructions. Primary antibodies: anti-TOMM20 (#ab78547, Abcam, Cambridge, UK), total OXPHOS rodent WB antibody cocktail (#ab110413, Abcam, Cambridge, UK), anti-vinculin (#ab18058, Abcam); secondary antibodies: HRP-conjugated goat anti-rabbit antibody (#AS014, Abclonal, Wuhan, China); HRP-conjugated goat anti-mouse antibody (#AS003, Abclonal). Stained protein bands were visualized using Clarity ECL kit (BioRad, Hercules, CA, USA) and Fusion FX gel-documentation system (Vilber Lourmat, Collegien, France) in the video mode to ensure that digital results were in the linear range. Optical density quantification was performed using the GelAnalyzer 19.1 software (www.gelanalyzer.com, accessed on 1 July 2021; software by Istvan Lazar Jr., PhD and Istvan Lazar Sr., PhD, CSc; Budapest, Hungary).

### 2.9. Statistical Analysis

Data is shown as mean ± standard error mean (SEM) for each group. Statistical analysis was performed using GraphPad Prism 8.0 software (GraphPad Software, San Diego, CA, USA). The significance of multiple differences (4 groups) was analyzed by the Kruskal–Wallis test with post-hoc Dunn’s test. The significance of paired differences (2 groups) was analyzed by the Mann–Whitney U-test. A statistical significance threshold of *p* < 0.05 was used in all mentioned statistical criteria.

## 3. Results

### 3.1. HGF/VEGF Plasmid Injection to Ischemic Muscle Aggravates Glucose Intolerance in HFD Conditions at 13 Week of Dietary Intervention

First, we analyzed the impact of *HGF/VEGF* plasmid injection into ischemic muscle on systemic metabolism parameters at the end of the post-surgery period (10 + 3 weeks) in HFD and LFD groups.

**Figure 1:**
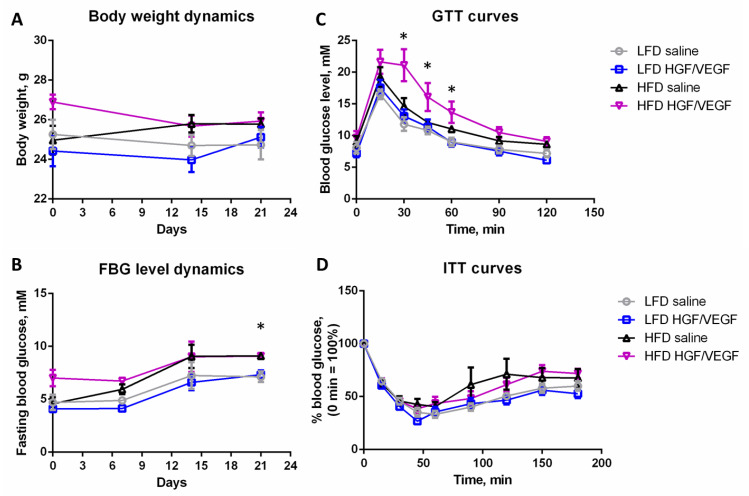
*HGF/VEGF* plasmid injection to ischemic muscle impairs glucose tolerance, but not other metabolic parameters, at 3 weeks after surgery (10 + 3 weeks of experiment). (**A**) body weight dynamics during post-surgical period; (**B**) FBG level dynamics during post-surgical period; (**C**) GTT curves on 10 + 3 weeks of experiment; (**D**) ITT curves on 10 + 3 weeks of experiment. Abbreviations: LFD, low fat diet; HFD, high fat diet; FBG, fasting blood glucose; GTT, glucose tolerance test; ITT, insulin tolerance test. Data is presented as mean ± SEM; Kruskal–Wallis test with post-hoc Dunn’s test; * *p* < 0.05.

Analysis of metabolic parameters demonstrated similar body weight dynamics without any statistical differences between groups (Figure 1A). FBG levels during the post-surgical period were variable yet *HGF/VEGF* plasmid injection did not affect normal glucose level in LFD conditions, but increased FBG level in HFD conditions (Figure 1B). GTT data analysis showed that in LFD conditions *HGF/VEGF* plasmid injection did not significantly change the GTT curve (Figure 1C). Nevertheless, *HGF/VEGF* plasmid injection to ischemic muscle seemed to disturb glucose tolerance in HFD conditions (Figure 1C). The ITT showed absence of significant changes in studied groups (Figure 1D). This data gave evidence that *HGF/VEGF* plasmid injection to ischemic muscle stimulates development of impaired glucose tolerance, but not insulin resistance without significant long-term metabolic consequences (FBG, body weight).

### 3.2. GTT Demonstrates a Shift to Glucose Intolerance after HGF/VEGF Plasmid Injection to Ischemic Muscle under HFD Conditions

Due to our finding that *HGF/VEGF* plasmid injection has an impact on glucose metabolism, we analyzed GTT curves obtained before surgery and at the experiments’ endpoint (10 + 3 weeks after *HGF/VEGF* plasmid or saline injection to ischemic hindlimb).

**Figure 2:**
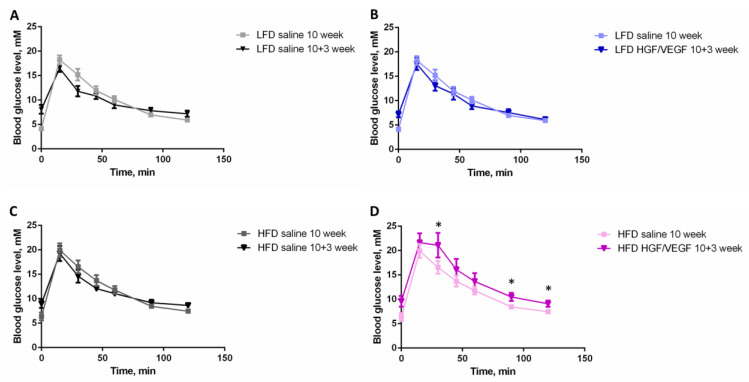
*HGF/VEGF* plasmid injection impairs glucose tolerance compared to pre-surgery state. (**A**) GTT curves for LFD group on 10 and 10 + 3 weeks of experiment; (**B**) GTT curves for LFD *HGF/VEGF* group on 10 and 10 + 3 weeks of experiment; (**C**) GTT curves for HFD group on 10 and 10 + 3 weeks of experiment; (**D**) GTT curves for HFD *HGF/VEGF* group on 10 and 10 + 3 weeks of experiment. Abbreviations: LFD, low fat diet; HFD, high fat diet; FBG, fasting blood glucose; GTT, glucose tolerance test. Data is presented as mean ± SEM, Mann–Whitney U-test; * *p* < 0.05.

This demonstrated that under the LFD condition neither surgery nor *HGF/VEGF* plasmid injection affected the GTT curve (Figure 2A,B). However, *HGF/VEGF* plasmid injection, but not surgery itself, impaired glucose tolerance in animals under HFD conditions (Figure 2C,D). ITT data analysis has demonstrated no influence of surgery or *HGF/VEGF* plasmid on ITT curves (Supplementary Figure S1A–D). Thus, *HGF/VEGF* plasmid injection to ischemic muscle during acute ischemia after surgery may aggravate impaired glucose tolerance under HFD, but not LFD conditions.

### 3.3. HGF/VEGF Plasmid Injection under HFD Conditions Results in Delayed Blood Flow Recovery with Similar Outcome at 3 Weeks in All Study Groups

After evaluation of the metabolic impact of *HGF/VEGF*, we estimated the effect of plasmid injection on ischemic limb blood flow recovery.

**Figure 3:**
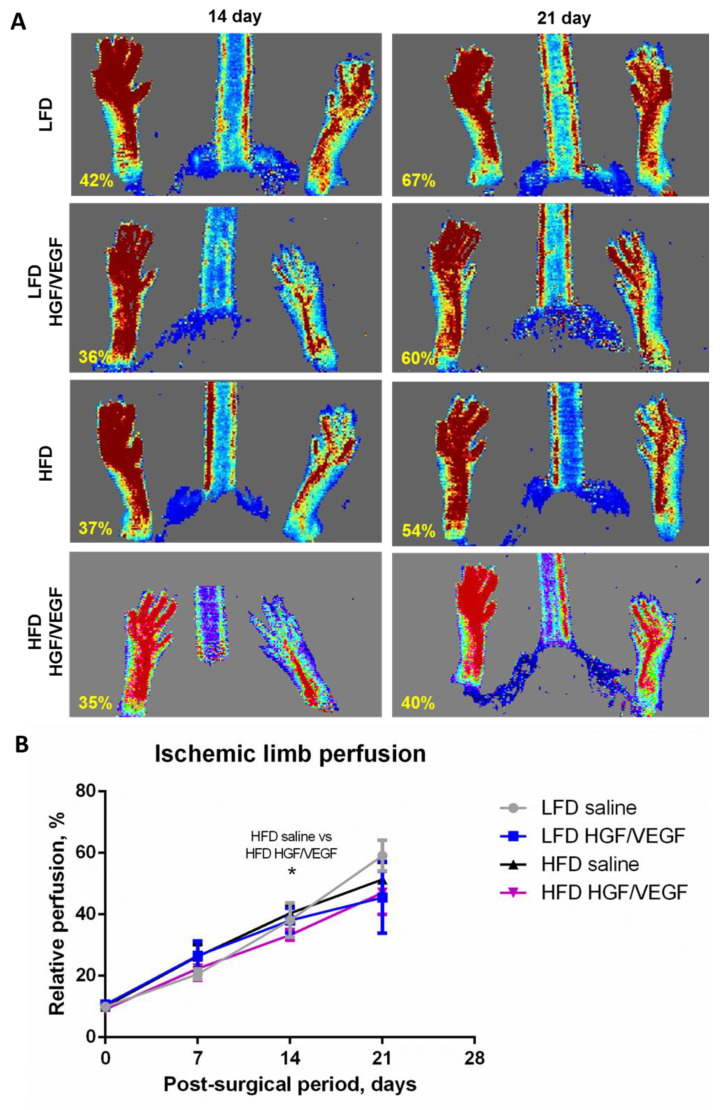
*HGF/VEGF* plasmid injection induces a transient delay of blood flow recovery in HFD group. (10 + 3 weeks of experiment). (**A**) representative Laser Doppler scanning images subcutaneous blood flow in experimental groups; (**B**) dynamics of blood flow recovery after *HGF/VEGF* therapy under LFD or HFD dietary intervention. Data is presented as mean ± SEM; Kruskal–Wallis test with post hoc Dunn’s test; * *p* < 0.05.

Analysis of blood flow curves in different groups showed that neither *HGF/VEGF* plasmid nor dietary intervention have a significant effect on the outcome - at 21 days all groups showed similar relative perfusion of the ischemic limb (Figure 3A,B). However, deeper analysis of intergroup differences demonstrated statistically significant changes in HFD group. Surprisingly, *HGF/VEGF* plasmid delayed blood flow recovery: at 14 days relative perfusion in the HFD saline group was higher than in the HFD *HGF/VEGF* plasmid group. Nevertheless, the final outcome values at 21 days were similar for all groups; this phenomenon needs additional discussion, as follows below.

### 3.4. HGF/VEGF Plasmid Injection had No Effects on Necrosis and Inflammation under HFD Conditions, but Increased These Parameters under LFD Conditions

To investigate possible mechanisms involved in delayed blood flow recovery under HFD conditions with *HGF/VEGF* plasmid injection we evaluated the necrotic area and CD68+ monocytes (predominantly, macrophages) infiltration in sections of ischemic *m. tibialis anterior* under different dietary and therapy conditions.

**Figure 4:**
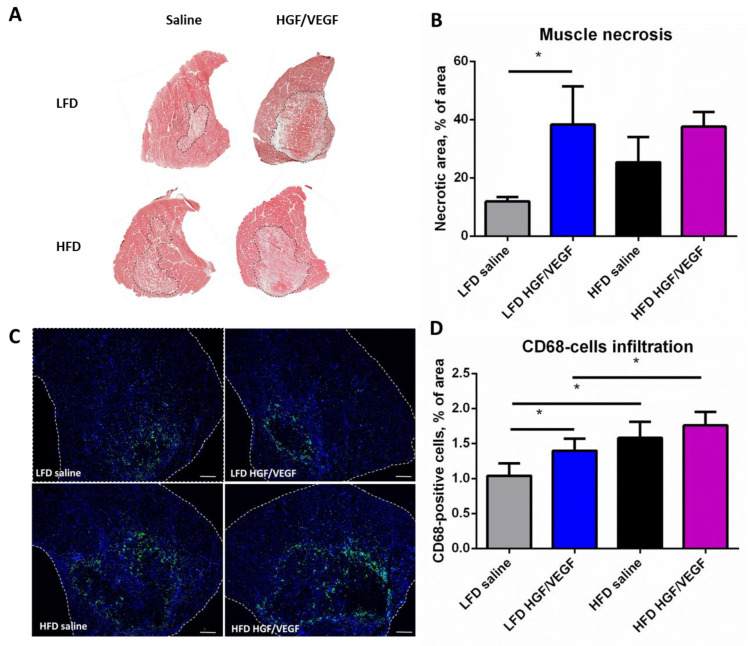
*HGF/VEGF* plasmid injection enhances ischemic-induced necrosis and macrophage infiltration under LFD conditions, but does not affect post-ischemic recovery process under HFD conditions. (10 + 3 weeks of experiment). (**A**) representative images of hematoxylin/eosin stained *m.tibialis anterior* sections, dotted line has marked necrotic area; (**B**) statistical analysis of necrotic area morphometry data; (**C**) representative images panel of CD68+ cells infiltration in *m.tibialis anterior*, scale bar 200 μm, dotted line has marked muscle area; (**D**) statistical analysis of CD68+ cells infiltration data. Data is presented as mean ± SEM; Kruskal–Wallis test with post hoc Dunn’s test; * *p* < 0.05.

We found that *HGF/VEGF* plasmid injection aggravated the span of necrosis in ischemic muscle in both LFD or HFD groups accompanied by the similar increment of CD68+ cells infiltration under LFD conditions (Figure 4A–D). This significant difference in necrotic area was of interest; we should notice that HFD itself did not cause significant enlargement of necrotic area (Figure 4A,B). However, HFD conditions significantly enhanced CD68+ cells accumulation in ischemic muscle both after saline or *HGF/VEGF* plasmid injections (Figure 4C,D). Despite the enlarged necrotic area and CD68+ cells accumulation, these changes did not compromise or improve subcutaneous blood flow recovery rate as shown in the previous subsection (Figure 3), which requires further discussion.

### 3.5. Delivery of HGF/VEGF Plasmid Fails to Improve Vascularization, but Activates Neuritogenesis under Both Dietary Settings

After analysis of ischemia-induced injury parameters, we studied possible angio- and neurogenic processes that can be induced by *HGF/VEGF* plasmid injection as both used growth factors are known to have angio- and neurogenic potency [23,24] widely implied in GT.

**Figure 5:**
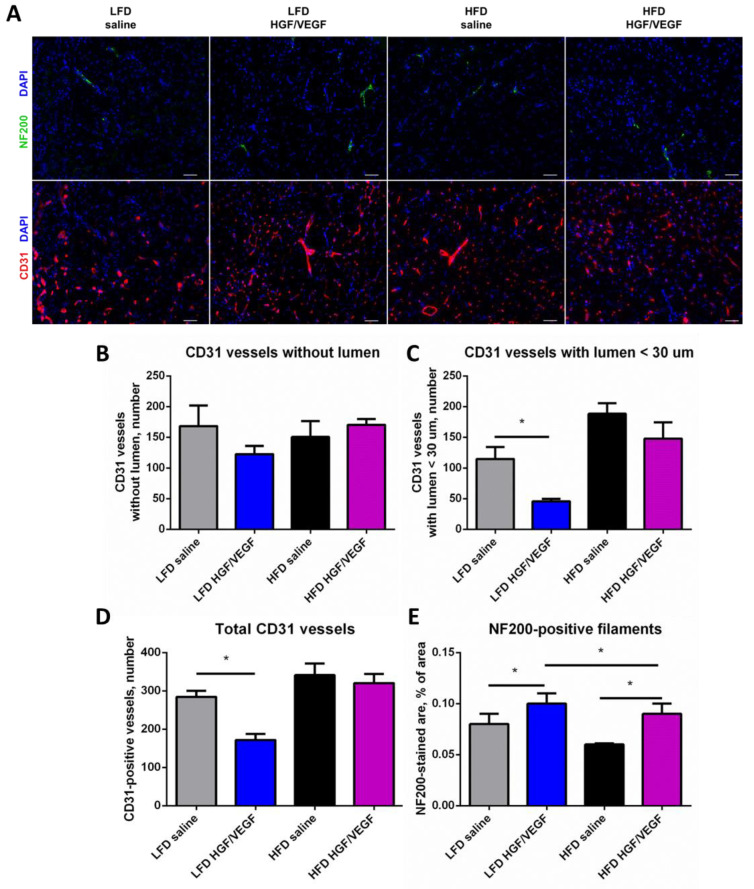
*HGF/VEGF* plasmid injection demonstrated limited impact on angiogenesis and activated both arteriogenesis and neuritogenesis under LFD conditions while under HFD was limited to neuritogenesis. (10 + 3 weeks of experiment). (**A**) representative images panel of NF200 and CD31 staining in *m.tibialis anterior*, scale bar 50 μm; (**B**–**D**) graphical presentation of blood vessel density analysis with average group values per field of view: CD31+ vessels without lumen (**B**), CD31+ vessels with lumen < 30 μm (**C**), CD31+ vessels with lumen >30 μm (**D**); (**E**) graphical presentation of neurites density analysis with average group values per field of view. Data is presented as mean ± SEM; Kruskal–Wallis test with post hoc Dunn’s test; * *p* < 0.05.

We found neither that diets nor plasmid delivery had effects on counts of capillaries without lumen (Figure 5A,B). However, *HGF/VEGF* plasmid injection demonstrated a surprising impact on capillaries with small (<30 μm) lumen: *HGF/VEGF* plasmid reduced the number of this capillary type under LFD conditions. Interestingly, this effect was absent in HFD conditions (Figure 5A,C), suggesting significant change of response due to dietary intervention. At the same time, the number of vessels with lumen >30 μm was significantly higher under LFD conditions after *HGF/VEGF* injection which was absent under HFD conditions (Figure 5A,D). Analysis of NF200+ structures in muscle sections at 3 weeks after injection demonstrated a statistically significant effect of *HGF/VEGF* plasmid, namely, increased NF200+ signal area which may reflect activation of neuritogenesis. This was observed both under LFD and HFD conditions and in the LFD GT group the increment was higher than in the HFD group (Figure 5A,E). In summary, *HGF/VEGF* plasmid injection exhibited a significant activating effect on neuritogenesis independently of dietary background, and LFD conditions favored a more potent response to *HGF/VEGF*.

### 3.6. HGF/VEGF Plasmid Injection Stimulated Mitochondrial Biogenesis and ETC Components Expression in Ischemic Skeletal Muscle under HFD Conditions

Finally, we analyzed the effects of *HGF/VEGF* plasmid under different dietary conditions on key bioenergetic properties of skeletal muscle tissue related to mitochondria content and electron transport chain (ETC) capacity. These parameters are crucial for skeletal muscle function as its mechanical work requires high ATP production and intensive oxidative metabolism and their recovery after ischemia are necessary for hindlimb recovery.

**Figure 6:**
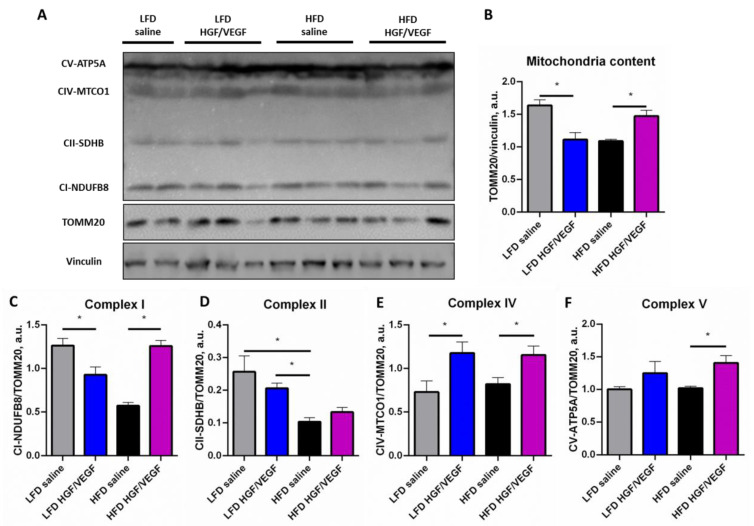
*HGF/VEGF* combined plasmid suppresses mitochondrial biogenesis and electron transport chain (ETC) input components expression in LFD conditions, but enhances these parameters in HFD conditions. (10 + 3 weeks of experiment). (**A**) representative Western blots panel; (**B**) mitochondria content in ischemic muscle; (**C**) NADPH-dehydrogenase (Complex I) expression level in ischemic muscle, (**D**) succinate dehydrogenase (Complex II) expression level in ischemic muscle; (**E**) cytochrome C oxidase (Complex IV) expression level in ischemic muscle; (**F**) ATP-synthase (Complex V) expression level in ischemic muscle. Data is presented as mean ± SEM, the Kruskal–Wallis test with post hoc Dunn’s test; * *p* < 0.05.

Analysis of energy supply systems in ischemic muscle after plasmid injection and under dietary intervention has been performed by evaluation of general mitochondrial marker (TOMM20) and ETC complexes content. First, it was shown that *HGF/VEGF* plasmid injection decreased general mitochondria contents under LFD conditions, whereas *HGF/VEGF* plasmid injection activated mitochondrial biogenesis under HFD conditions (Figure 6A,B). Moreover, analysis of ETC complexes normalized to total mitochondria demonstrated that *HGF/VEGF* plasmid injection to ischemic muscle suppressed the level of complex I which can be related with reduction of NADH input to ETC (Figure 6A,C). After plasmid injection, complex IV contents were enhanced under LFD conditions as well (Figure 6A,E). However, under HFD conditions in ischemic muscle, *HGF/VEGF* plasmid rendered a synergistic effect: levels of complexes I, IV and V were significantly increased compared to saline control (Figure 6A,C,E,F). This may suggest that in ischemic muscle *HGF/VEGF* plasmid injection promotes recovery of ATP synthesis, crucial for motor activity and survival of muscle.

## 4. Discussion

We have shown that HFD effectively induced impaired glucose tolerance which was confirmed by a disturbed GTT response (Figure 1C) accompanied by a normal ITT curve (Figure 1D). It was an expected reaction for mice on high dietary intake of saturated fat while we may suggest that the HFD duration was insufficient for full insulin resistance onset. However, the effect of *HGF/VEGF* plasmid was surprising: under LFD conditions *HGF/VEGF* plasmid did not affect the GTT curve, but in HFD conditions the combined *HGF/VEGF* plasmid caused impaired glucose tolerance. Traditionally, *HGF* and *VEGF165* are characterized as insulin-sensitizing growth factors [25,26,27]. Nevertheless, if we look at insulin resistance (or impaired glucose tolerance) not as a pathological status, but as a physiological adaptation to alimentary conditions, we may suggest that changes in glucose tolerance induced by *HGF/VEGF* GT reflect enhancement of energy supply for tissue recovery. Such putative plasmid action in HFD conditions (known to suppress regenerative processes) can be potentially beneficial for ischemic skeletal muscle recovery. Similar cases of physiological adaptation are well-known: e.g., insulin resistance during pregnancy or post-surgical insulin resistance aimed to maximize energy supply of fetus or body recovery, respectively [28,29]. This was confirmed by data in Figure 2 as well, where administration of *HGF/VEGF* plasmid in HFD animals enlarged the AUC in GTT after surgery when compared to the state before the induction of ischemia. Thus, delivery of *HGF/VEGF* via GT may have an adaptive impact on systemic metabolism in both healthy mice and animals with hindlimb ischemia.

Evaluation of efficacy in GT designed to stimulate blood flow recovery relies on a wide array of methods, and laser Doppler assessment is a well established approach in small animal models [30]. In our work we were definitely surprised to see the impact of dietary intervention and lack of *pHGF/VEGF* efficacy (Figure 3) with a transient delay at 14 days in the HFD group with plasmid injection compared to HFD saline-treated control. From a clinical point of view this correlated with our findings in GTT where *pHGF/VEGF* injection has aggravated impaired glucose tolerance (Figure 2). Indeed, the clinical relevance of this finding is much supported by data from patients that demonstrated negative trends of PAD prognosis once diabetes mellitus was diagnosed [31]. At the endpoint (21 days), laser Doppler perfusion curves ended with a similar relative perfusion with no statistical significance between any pair of groups. Such dynamics contrast with our previous data in animals without impairment of glucose metabolism (fed by standard grain-based chow). In our first work, a mixture of two pDNAs with *HGF* or *VEGF165* gene has shown potent blood flow increase in ischemic muscle [9]. Furthermore, the same bicistronic *HGF/VEGF* plasmid has been tested in mice on grain-based chow and has shown high angiogenic potency [8] which failed to reproduce in both LFD and HFD groups of the present study.

This drop of efficacy demonstrates that dietary intervention (both-LFD or HFD) has a great impact on the pharmacological activity of a non-viral GT drug designed to stimulate reperfusion. We can hardly draw a definite conclusion on the mechanisms of this finding yet *HGF* and *VEGF* expression should be evaluated in LFD or HFD settings. We found no experimental works on how glucose tolerance impairment might affect pDNA GT kinetics and feel this is an issue to be addressed further.

Histology data was also discrepant with our and other groups’ findings on how GT might impact necrosis and CD68+ inflammatory cells infiltration. We found that after GT by *HGF/VEGF* in LFD groups muscle necrosis was increased compared to saline-treated animals; a similar trend was observed in the HFD group treated by GT (Figure 4A,B). This might be explained by a non-specific pro-inflammatory effect by large amounts of pDNA injected to the muscle known to activate toll-like receptors (TLRs) and promote monocyte invasion [32]. Previously, due to presence on CpG motifs even empty pDNA backbones (without and cDNA or genomic sequence within) have been shown to drive inflammation [33] and even slightly increase necrotic area in mouse hind limb ischemia compared to vehicle injection [8]. Whether this is a relevant explanation given the 21 days of experiment before the tissue samples were isolated is an open question. Nevertheless, dietary interventions have shown a potent negative influence on *pHGF/VEGF* ability to recover blood flow and consequently-prevent muscle necrosis. However, under LFD conditions this parameter seems more sensitive with significant increase of necrosis probably due to a lower basal level of muscle damage compared to HFD animals. In the latter, glucose tolerance impairment may aggravate the initial ischemic injury to muscle tissue and increase necrosis in saline-treated mice, masking the effects of *HGF/VEGF* administration (Figure 4B).

*VEGF165* and *HGF* are known to be counteracting in terms of CD68 cells attraction as far as VEGFR2-mediated activation of NF-kB and inflammatory cytokines might be mitigated by activation of c-met by HGF known to downregulate NFkB [34]. However, in our study we found that in LFD settings *HGF/VEGF* induced invasion of additional monocytes while in HFD it has failed to promote their chemotaxis (Figure 4C,D). As for higher numbers of CD68 cells in both HFD vs. LFD comparisons (LFD-saline vs. HFD saline and LFD *HGF/VEGF* vs. HFD *HGF/VEGF*), this was a relevant indicator of potent pro-inflammatory response to pDNA correlating with necrosis span (Figure 4B) which drives invasion of phagocytes to clear the debris.

Besides painful necrosis (ulcers and gangrene), patients with advanced PAD suffer from a combined impairment of tissue tropics induced by low oxygen supply and, furthermore, neuropathic changes that lead to even worse clinical prognosis [35]. Prevalence of neuropathy is increased in diabetic patients with PAD and has also become a driver for GT development to treat this condition characterized by intense pain [36]. This prompted us to evaluate both blood vessel counts and axonal density in sections of muscle injected by *pHGF/VEGF* or saline. During angiogenesis assessment we encountered a contrasting picture in LFD and HFD subgroups and a drop of general vascularization (total CD31, Figure 5D) was found in LFD while in HFD this parameter was not changed by GT. This seems to have been much due to vessels with small (<30 μm) lumen (Figure 5C) known to play a pivotal role in blood supply. This might be explained by either remodeling of early-formed blood vessels by 21 days of experiment or (which is more likely) that under LFD conditions animals may suffer from impaired angiogenesis due to ongoing necrosis (Figure 4B) which was very prominent in the *pHGF/VEGF* group. Overall, blood vessel counts did not demonstrate any increase after *pHGF/VEGF* which correlated with Doppler perfusion assessment failing to detect blood flow improvement (Figure 3).

Eventually, we found that structures positive for NF200 (axonal marker) evaluated by area in sections was the only physiologically relevant parameter that responded to stimulation by *pHGF/VEGF* and improvement in LFD group was more prominent that in HFD settings (Figure 5E). While this might look as a “drop in the sea” positive finding in this study, it puts an important stress on a relevant clinical problem that might be addressed by *pHGF/VEGF* treatment. *VEGF165* and *HGF* both have been shown to have a neurotrophic modality via corresponding signaling axes [37,38] and this has become the basis for their application in peripheral nerve injury, central nerve system diseases and neuropathic conditions [39,40]. There is a global unmet medical need in diabetic patients with/without PAD for non-viral GT to be developed further. Fully understanding the limitations of *pHGF/VEGF* neurotrophic activity statements due to the absence of functional models, we still may suggest that this can be the direction for further development and designation for this prospective drug.

Finally, intrigued by the nature of GTT changes induced by *HGF/VEGF* in HFD group (Figure 1) we performed a set of experiments assessing metabolic status in regenerating ischemic muscle discussed below.

Analysis of mitochondria in muscle samples isolated at endpoint (21 days after surgery) revealed a decrease of mitochondria content under HFD conditions in the saline control group (Figure 6A,B) which is in line with the stipulated metabolic adaptation of muscle cells to nutrients overload [41,42]. The injection of *HGF/VEGF* plasmid improved this effect; the mitochondria content of GT-treated ischemic skeletal muscle was higher than in the saline group (Figure 6A,B). In contrast to the above mentioned effect, *HGF/VEGF* plasmid delivered under LFD conditions lowered mitochondria content in ischemic skeletal muscle (Figure 6A,B). From a mechanistic point of view, this can be explained by the effects of *HGF* and *VEGF165* on skeletal muscle mitochondria. Recent studies have shown that VEGF can enhance the oxidative balance of mitochondria without changes in Krebs cycle enzymes activity and mitochondrial biogenesis factors [43]. Published data about the effects of HGF on mitochondria content in skeletal muscle is scarce. It has been shown that HGF is involved in mitochondrial function in stem cells, but certain studies report that HGF may increase the rate of glucose metabolism along with suppression of fatty acid oxidation in skeletal muscle [44,45]. The latter can be an indicator of mitophagy and possible decrease of mitochondria content.

GT-dependent growth of mitochondria content in the HFD group can be related to the systemic action of *HGF/VEGF* GT due to delivered growth factor circulation which was observed in clinical trials of pDNA drugs [45]. However, our data on the local effects of *HGF/VEGF* plasmid in HFD animals suggests that a local shift of metabolism may impact other tissues and thus impair glucose tolerance which we observed in GTT. Indeed, in a physiological state, insulin suppresses lipolysis in adipose tissue, but through adaptive insulin resistance *HGF/VEGF* expression can decrease the suppressive action of insulin on lipolysis and thus increase fatty acid plasma concentration. Circulating fatty acids are a potent activator of muscle mitochondria biogenesis [46] and this mechanism potentially links *HGF/VEGF* plasmid impact on systemic metabolism (GTT) and mitochondria content in muscle.

We also performed a thorough analysis of ETC components content in ischemic skeletal muscle after plasmid GT. It is well known that complex I and complex II are inputs of ETC where the energy of NADH and FADH2 transforms into a proton gradient. Complex IV also is an important part of the proton gradient-generating system. Complex V is ATP-synthase which performs the energy supply for skeletal muscle contractility. We have demonstrated that contents of ETC input components declined under HFD conditions which strongly correlated with mitochondrial contents data and supported the hypothesis about adaptive reaction of skeletal muscle mitochondria to HFD. However, *HGF/VEGF* plasmid therapy of ischemic muscle in HFD significantly increased the content of complexes I, IV and V. This suggests not simply increased mitochondria contents, but an increased proportion of energetically active mitochondria readily transforming NADH to energy at a significantly higher rate. This can be a very beneficial point for formation of new vessels, axons, myofibers and regenerative processes in general. As for reference studies in the field, we seem to be the first group to address the muscle bioenergetic shifts after *HGF* and *VEGF165* gene therapy as we found no published data on this subject.

## 5. Limitations

A limitation of our study could be a non-optimal time of *HGF/VEGF* plasmid injection. The time course of ischemic injury includes an acute inflammation period (day 3–7) and resolution period (resolution of inflammation, angiogenesis, blood flow recovery; (day 7–21). The acute inflammation period may not be the best time for the *HGF/VEGF* plasmid administration. However, we took into account with our previous results [8], where *HGF/VEGF* plasmid injection during hind limb ischemia modeling had a significant effect on subcutaneous blood flow recovery, inflammation and necrosis reduction; in the case of dietary intervention, the time point of *HGF/VEGF* plasmid administration may be critical for outcomes.

## 6. Conclusions

The current study demonstrated a significant role of dietary conditions in pre-clinical testing of non-viral GT drugs. We also may conclude that angiogenesis and blood flow are extremely sensitive (at least in small rodents) to HFD and LFD and even a potent GT drug like *pHGF/VEGF* may show a great drop of angiogenic efficacy despite our previous data for standard dietary conditions. However, this decrease of potency is differential and we found that innervation was recovered by *HGF/VEGF* in the HFD group. We thus believe that our work is a contribution demonstrating the importance of relevant preclinical modeling and describing the impact of dietary interventions in mice as the most widely used species in studies of GT to treat PAD.

As for novelty in terms of GT, our analyses of bioenergetics and GTT allow us to suggest (with a certain degree of speculation) that especially in HFD conditions *HGF/VEGF* plasmid gene therapy of hindlimb ischemia can cause adaptive insulin resistance which induces mitochondrial biogenesis in skeletal muscle with consequent enhancing of regeneration energy supply. This could make the *HGF/VEGF* combined plasmid a very promising tool for PAD therapy in obese and diabetic conditions.

## Data Availability

The raw data supporting the conclusions of this article will be made available by the authors, without undue reservation.

## Supplementary Materials

The following supporting information can be downloaded at: https://www.mdpi.com/article/10.3390/cells11233824/s1, Figure S1: HGF/VEGF plasmid injection did not affect insulin-dependent glucose clearance in comparison with pre-surgery state.

## Abbreviations

ATP—adenosine triphosphate; BSA—bovine serum albumin; CD31—cluster of differentiation 31 or platelet endothelial cell adhesion molecule (PECAM-1); CD68—cluster of differentiation 68 or macrosialin; ETC—electron transport chain; FBG—fasting blood glucose; GT—gene therapy; GTT—glucose tolerance test; HGF—hepatocyte growth factor; HFD—high fat diet; HRP—horseradish peroxidase; ITT—insulin tolerance test; LFD—low fat diet; NF200—neurofilament heavy chain 200 kDa; OXPHOS—oxidative phosphorylation metabolism; pDNA—plasmid DNA; PAD—peripheral artery disease; PBS—phosphate buffer solution or saline; SDS PAGE—sodium dodecyl sulfate supplemented polyacrylamide gel-electrophoresis; SEM—standard error of mean; T2DM—type 2 diabetes mellitus; VEGF—vascular endothelial growth factor.
