# Supplementary material for: The Efficacy of HGF/VEGF Gene Therapy for Limb Ischemia in Mice with Impaired Glucose Tolerance: Shift from Angiogenesis to Axonal Growth and Oxidative Potential in Skeletal Muscle
Source: Cells. 2022 Nov 29;11(23):3824. doi: 10.3390/cells11233824 (PMC9737863; doi:10.3390/cells11233824)
Supplement: Supplementary file 1 [file cells-11-03824-s001.zip › cells-2043177-supplementary.pdf]

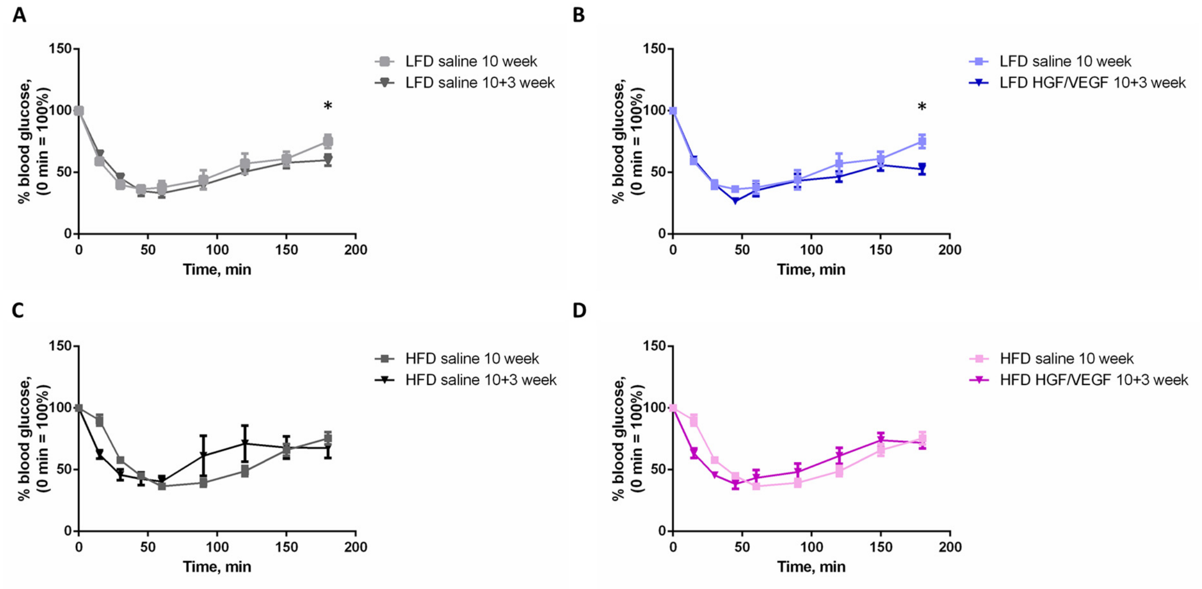

Supplementary Figure 1. HGF/VEGF plasmid injection did not affect insulin-dependent glucose clearance in comparison with pre-surgery state. Abbreviations: LFD, low fat diet; HFD, high fat diet; GBD, grain based diet; FBG, fasting blood glucose; GTT, glucose tolerance test; ITT, insulin tolerance test. Data are represented as mean ± SEM, Kruskel-Wallis test with post-hoc Dunn's test, significance threshold $p < 0.05$
